# A function‐valued trait approach to estimating the genetic basis of size at age and its potential role in fisheries‐induced evolution

**DOI:** 10.1111/eva.12771

**Published:** 2019-02-27

**Authors:** Jin Gao, Stephan B. Munch

**Affiliations:** ^1^ Department of Ecology and Evolution Stony Brook University Stony Brook New York USA; ^2^ School of Marine and Atmospheric Sciences Stony Brook University Stony Brook New York USA; ^3^ Present address: Centre for Fisheries Ecosystems Research Fisheries and Marine Institute of Memorial University of Newfoundland St. John's Newfoundland and Labrador, Canada; ^4^ Present address: National Marine Fisheries Service Southwest Fisheries Science Center Santa Cruz California USA

**Keywords:** fisheries‐induced evolution, function‐valued trait, G matrix, growth rate, life history, selectivity

## Abstract

Natural selection is inherently a multivariate phenomenon. The selection pressure on size (natural and artificial) and the age at which selection occurs is likely to induce evolutionary changes in growth rates across the entire life history. However, the covariance structure that will determine the path of evolution for size at age has been studied in only a few fish species. We therefore estimated the genetic covariance function for size throughout ontogeny using Atlantic silversides (*Menidia menidia*) as the model system. Over a 3‐year period, a total of 542 families were used to estimate the genetic covariance in length at age from hatch through maturity. The function‐valued trait approach was employed to estimate the genetic covariance functions. A Bayesian hierarchical model was used to account for the unbalanced design, unequal measurement intervals, unequal sample sizes, and family‐aggregated data. To improve mixing, we developed a two‐stage sampler using a Gibbs sampler to generate the posterior of a well‐mixing approximate model followed by an importance sampler to draw samples from posterior of the completely specified model. We found that heritability of length is age‐specific and there are strong genetic correlations in length across ages that last 30 days or more. We used these estimates in a hypothetical model predicting the evolutionary response to harvesting following a single generation of selection under both sigmoidal and unimodal patterns of gear selectivity to illustrate the potential outcomes of ignoring the genetic correlations. In these scenarios, genetic correlations were found to have a strong effect on both the direction and magnitude of the response to harvest selection.

## 1 INTRODUCTION

The effects of harvest can lead to important, potentially irreversible, evolutionary changes in life‐history characteristics (Haugen & Vøllestad, 2001; Conover & Munch, 2002; Law, 2007; Enberg et al., 2011). Fishing reduces overall survival to later life stages and typically removes larger individuals, thereby selecting for earlier maturation and smaller adult size (Law, 2000). At the same time, natural selection favoring larger fish is very strong, particularly during the early life history (Perez & Munch, 2010), leading to the hypothesis that larval fish should grow as fast as possible (Leggett & Deblois, 1994). Thus, the rate and direction of fishery‐induced evolution will depend on the balance of these opposing forces of selection, the amount of standing genetic variation in the population, and the degree to which size early and late in life are genetically correlated.

Knowledge of both the phenotypic and genetic aspects of variation is essential to understanding the potential for contemporary evolution (Kirkpatrick & Lofsvold, 1992; Grant & Grant, 1995). Heritability, or the ratio of additive genetic variation to total phenotypic variation, is widely used as an index of evolutionary potential. Scalar estimates of heritability for size at age in fishes range widely (reviewed by Gjedrem, 1983; Law, 2000) but tend to cluster around 0.26, similar to other important life‐history traits (Mousseau & Roff, 1987). Although studies of fisheries‐induced evolution often assume a single value for heritability, these estimates may be highly age‐specific. For example, in rainbow trout (*Salmo gairdneri*), heritability for length and weight ranged from 0.13 at age 2.5 years to 0.38 at age 4 years (McKay, Ihssen, & Friars, 1986). In Atlantic silversides, heritability of size at age is 0.1 at hatch and increases to 0.25 by age 10 days (Gao & Munch, 2013). Although the heritability of size in fishes tends to be fairly modest, there is clearly a substantial genetic component to growth and we should expect size trajectories to respond to harvest selection.

Models of fisheries‐induced evolution have typically used a specific function relating size and age (e.g., von Bertalanffy). When growth is allowed to evolve in these models, it is usually a single parameter that evolves rather than the shape of the growth trajectory (e.g., Andersen & Brander, 2009). Implicit in this approach are the assumptions that the shape of growth trajectories is highly constrained and that selection on size at one age will affect size at all other ages. The adequacy of these assumptions is not well tested, but could be addressed by measuring the genetic correlations between size at different ages. When genetic correlations are weak or absent, harvest selection can only be opposed by natural selection acting at the same age.

Moreover, genetic correlations can place constraints on evolution beyond those implied by a single heritability estimate (Blows & Hoffmann, 2005; Kirkpatrick, 2009). For example, in rainbow trout, genetic correlations in body weight between different ages are positive and range from 0.57 to 0.93, decreasing with the interval between two ages (Su, Liljedahl, & Gall, 2002). These correlations are substantial enough that the response to harvest selection will depend on the balance of selection across multiple ages. In this case, scalar estimates of heritability would be insufficient to predict either the rate or direction of evolution.

One approach to modeling the influence of genetic correlations on the evolution of size at age in harvested populations might be to treat size at a set of discrete ages as a vector‐valued trait. In studies of multivariate evolution, the additive genetic covariance matrix, ***G***, conveniently summarizes the genetic relationships among a suite of traits and is a central parameter in determining response to selection (Lande, 1979; Agrawal, Brodie, & Rieseberg, 2001). For a vector‐valued trait, the predicted response to selection is a multivariate generalization of the breeders’ equation: the change in mean trait values ($\overset{¯}{Z}$) in the next generation is given by $\Delta \overset{¯}{Z}=G\beta$, where $\beta =\nabla_{\overset{¯}{Z}}\text{ln}\left[\overset{¯}{W}\right]$ is the selection gradient vector of mean fitness with respect to the trait mean (Lande, 1979; Lynch & Walsh, 1998). At a specific age, say *t*_*i*_ *,* the predicted response to selection is $\Delta \overset{¯}{Z}\left(t_{i}\right)=G_{i,i}\beta_{i}+\sum_{j\neq i}G_{i,j}\beta_{j}$, which is the sum of the direct response and the indirect effects of selection on other ages. In the presence of strong genetic correlations, or a large number of correlated traits, the indirect effects can outweigh the direct response. In this case, predicting evolution from a scalar heritability estimate may underestimate the rate of change or even get the sign wrong.

Despite the potential utility of a multivariate approach, age is really continuous and discretizing age to obtain a vector‐valued trait may introduce artifacts. Genetic variation in traits with a continuous index such as age can instead be modeled via a function‐valued trait approach (FVT) (Griswold, Gomulkiewicz, Heckman, & Promislow, 2008; Kingsolver & Gomulkiewicz, 2003; Kirkpatrick & Lofsvold, 1992; Stinchcombe & Kirkpatrick, 2012), which is a continuous generalization of classical multivariate methods (Lande, 1979). The FVT approach treats size as function of age and therefore attempts to estimate a covariance *function* rather than a covariance matrix. For classical multivariate traits, there is no *a priori* structure to the dependence among size at each age. In contrast, continuity and smoothness require that the sizes at nearby ages be highly correlated. Thus, one of the main advantages of the FVT approach is that it retains information about the ordering and spacing of a set of data points, while this information is ignored in a classical multivariate analysis.

The FVT approach has been applied in many areas of evolutionary ecology (reviewed by Stinchcombe & Kirkpatrick, 2012). Some examples include the study the evolution of reproduction and mortality trajectories in *Drosophila* (Jaffrezic, Thompson, & Hill, 2003), the evolution of thermal performance curves in caterpillars (Izem & Kingsolver, 2005), and the evolution of growth trajectories in lizards (Ragland & Carter, 2004) and fishes (Kirkpatrick & Lofsvold, 1992). Thus, the FVT framework can, in principle, be used to predict the evolutionary effects of harvesting on growth (for a theoretical treatment see, e.g., Dieckmann, Heino, & Parvinen, 2006). However, in order to do so in practical application, we need an estimate of the genetic covariance function. To our knowledge, no study has estimated the *G* function across the entire range of life stages in a fish species.

We used Atlantic silversides (*Menidia menidia*) as a model organism to investigate the genetic basis of growth trajectories over the entire growing season from birth through maturation. *M. menidia* is an annual fish (Conover & Ross, 1982) commonly found from northeast Florida to the Gulf of St. Lawrence (Johnson, 1974). Over this range, silversides exhibit countergradient variation in growth (Present & Conover, 1992; Hice, Duffy, Munch, & Conover, 2012). The heritability of size at maturity is fairly high (~0.2), at least for fish from New York (Conover & Munch, 2002). Although Munch, Walsh, and Conover (2005) estimated realized co‐heritability for size from hatch to 190 days, there is as yet no estimate of the genetic covariance function for size in *M*. *menidia*.

In this study, we developed a FVT trait approach to model the genetics of size at age in Atlantic silversides. To do so, we apply a FVT analysis based on the classical animal model (Lynch & Walsh, 1998). We use the results to calculate the heritability of length at age using estimated genetic and phenotypic covariance functions. By partitioning variance among sires and dams separately, we estimated the age dependence of maternal contributions to size. Finally, to illustrate the value of the FVT approach for fisheries‐induced evolution, we use our results to predict the response to size‐selective harvest under two hypothetical patterns of gear selectivity. To evaluate the importance of genetic correlations for fisheries‐induced evolution, we evaluated the response to selection under two alternative models for the genetic covariance of length at age.

## 2 METHODS

We begin by describing the model organism and the experiments conducted to estimate the genetic contributions to size at age. Following this, we introduce the quantitative genetic model that we used to analyze the size at age data. Finally, we insert these results into a model to predict the evolutionary response to size‐selective harvesting.

### 2.1 Study system

Atlantic silversides were collected during the peak of the breeding season (end of May to beginning of June) from two sites on the north shore of Long Island (Poquott, East Setauket, New York, and Flax Pond, Old Field, New York; 40°57′49″ N, 73°8′19″ W) and two south shore sites (Great South Bay and Shinnecock Bay, New York; 40°51′10″ N, 72°29′27″ W). The north shore and south shore sites exchange only a limited number of migrants each year (Clarke, Munch, Thorrold, & Conover, 2010), ensuring that the parents from the disparate locations are at most distantly related. Adults were transported back to the Flax Pond Marine Lab, Old Field, New York (FPML), where they were housed overnight in separate tanks and strip‐spawned the following day.

Because space constraints prevented us from rearing >500 families simultaneously, the study was carried out in three batches using adults collected on May 1, 2008, June 1, 2008, and May 24, 2009. The first round of experiment was focused on estimating genetic and maternal contributions to the early life history and lasted 15 days (Gao & Munch, 2013). Batches 2 and 3 were maintained for 176 and 273 days, respectively. As described below, the data from these three batches are combined in a Bayesian analysis, which accounts for batch effects separately in each round. Each of the three spawning batches consisted of several complete‐factorial blocks. To limit the relatedness among parents, the north shore males were only mated to the south shore females (and vice versa) and no parents were used in more than one block. Because the numbers of fish of each sex captured in each field collection were beyond our control, there were differences in the breeding design among batches. Table 1 reports the number of sires and dams per block for each batch as well as the total number of blocks per batch. Note that the total number of families (Table 1) analyzed is always less than the maximum (sires × dams × blocks) due to unsuccessful spawning or limited hatching and rearing success.

**Table 1:** The breeding design and sample sizes for each of the three spawning batches

Batch	*N*_*s*_	*N*_*d*_	*N*_*b*_	*F*	*t*
1	3	4	10	97	4
2	3	10	5	147	16
3	3	5	11	133	11

*N*_*s*_ and *N*_*d*_ are the numbers of sires and dams used to construct each complete‐factorial block. *N*_*b*_ is the total number of blocks reared in the batch, and *F* is the number of surviving families that are included in the analysis. The number of total families for each family is indicated by *t*.

To create each family block, eggs were stripped from a female and distributed across several Petri dishes lined with fiberglass screening and a shallow layer of seawater. At the same time, milt from each male was stripped into a small beaker and diluted with UV‐sterilized seawater. Milt from each sire was then distributed among the Petri dishes for each female, such that within a block all males were mated with all females. After allowing 20 min for the fertilized eggs to harden, eggs from each family were transferred to an aerated 18‐L bucket immersed in a previously designated seawater bath. To avoid possible confounding of the family and bath effects, families were assigned to baths in a stratified‐random manner such that each family block was guaranteed to occur in multiple baths.

At 15 days posthatch, the fish in each family were split into two replicates with 30 fish in each replicate and assigned to different baths. Once the fish reached ~25 mm in standard length (roughly 30 days posthatch), each individual was injected with a Visible Implant Elastomer Tag (Northwest Marine Technology) underneath the skin adjacent to the dorsal fin. After tagging, fish from each family were subdivided into two groups and transferred into randomly selected 3,785‐L polyethylene tanks. There were 15 such tanks in total, and each tank contained individuals from ~9 families. As with sea tables, tank assignments were stratified by family such that no family occurred in only one tank allowing us to separate family and tank effects. Overall, 542 families of fish were reared over the 3‐year period.

Throughout the experiment, the seawater was maintained at 21°C (±1.2°C). During the larval period, fish were fed to satiation daily using a combination of dry feed (Otohime larval feeds, Reed Mariculture) and freshly hatched *Artemia* nauplii (Brine Shrimp Direct). During the juvenile and adult stages, fish were fed a combination of dry food (Otohime juvenile and adult feeds, Reed Mariculture) and frozen adult brine shrimp. Further details of the rearing protocol are described in Present and Conover (1992).

### 2.2 Length measurements

During the larval stages from days 1 to 15, the fish were measured using several approaches, including digital photography (batches 1 and 2) and calipers (batch 3). Repeated measurements of 100 individuals indicated that there are no systematic biases in any of the measuring approaches and that they each had comparable levels of precision. During the juvenile and adult stages, the lengths all of the fish in each replicate were estimated using digital photography. Specifically, a 100‐megapixel digital camera (Canon 40D with Canon 60 mm Macro lens) was used to photograph the fish from a fixed height at a shutter speed of 1/250 s. Larvae were held in a Petri dish with a shallow layer of water and photographed from 55 cm. Juveniles and adults were held in an 18‐L bucket with a 4 cm layer of water and photographed from a fixed height of 140 cm. The images were then measured in Image Pro Plus 6.0 (Media Cybernetics).

### 2.3 Statistical analysis

The results of the three rounds of experiments were combined via Bayesian modeling. Silversides are an obligate schooling fish that require ample swimming space and do not grow normally in isolation. However, space constraints limited the number of tanks we could use and several hundred fish were reared in each tank. Since only eight tag colors were available, individual fish could not be tracked throughout the study. Therefore, although we have sizes of each fish in each family at every measurement, we have no way to rigorously connect individual identities through time. In addition, not all individuals survived to the end of the experiment so that the numbers of individuals in each family were not constant. To circumvent these difficulties, we develop novel methods for estimating the genetic covariance function from these family‐level data (see Supporting Information). We note that this is, in effect, a repeated‐measures design with longitudinal data for each family. An appropriate statistical framework for such data must account for persistent differences among families, which is captured in the function‐valued trait approach.

### 2.4 FVT analysis

The classic animal model (Lynch & Walsh, 1998) uses a linear combination of fixed, genetic, and environmental effects to approximate the trait value. In keeping with the literature on function‐valued traits, we assume that an individual's growth trajectory can be described as

(1) $$y_{i}\left(t\right)=\mu \left(t\right)+\beta_{i}\left(t\right)+\frac{1}{2}\left[g_{\mathrm{di}}\left(t\right)+g_{\mathrm{si}}\left(t\right)\right]+\epsilon_{i}\left(t\right)$$

where μ is the grand mean growth trajectory and β_*i*_ represents the fixed effect of either sea table (ages 0–30 days) or tank (ages 30–276 days) estimated independently at each time point. The grand mean and fixed effect functions were piecewise linear, effectively taking independent values at each age for which we had data. The *g*_*s*_ and *g*_*d*_ functions represent the genetic contributions of the sire and dam, respectively, and ဓ represents the unexplained “environmental” contributions to size.

Using the silverside data, our main interest is in estimating the genetic covariance function for sires, $C_{s}\left(t,t^{\prime }\right)=E\left[g_{s}\left(t\right)g_{s}\left(t^{\prime }\right)\right]$ and the environmental covariance function, $C_{\epsilon }\left(t,t^{\prime }\right)=E\left[\epsilon \left(t\right)\epsilon \left(t^{\prime }\right)\right]$. The covariance function for dams, $C_{d}\left(t,t^{\prime }\right)=E\left[g_{d}\left(t\right)g_{d}\left(t^{\prime }\right)\right]$, is also of interest but includes both genetic and maternal effect contributions to offspring (Lynch & Walsh, 1998).

The parental effects are modeled as Gaussian processes using a Legendre polynomial basis expansion. Specifically, $g_{s}\left(t\right)=\sum_{k=1}^{K}h_{k}\left(t\right)\gamma_{s,k}$ and $g_{d}\left(t\right)=\sum_{k=1}^{K}h_{k}\left(t\right)\gamma_{d,k}$ where the *h*_*k*_s are the polynomial basis functions and the γs are the basis expansion coefficients (To avoid confusion with the heritability which is traditionally denoted *h* ^2^, basis functions will always have a subscript). The environmental deviation term is modeled similarly, using the same basis set. Note that the basis expansion approach to modeling a function‐valued trait is equivalent to a classical multivariate approach, albeit in the abstract “trait space” of the basis coefficients. As such, the basis coefficients are assigned their own covariance matrices, which we denote by **C**_**s**_, **C**_**d**_, and **E** for the genetic contributions of sires, dams, and the environmental basis coefficients. The number of basis functions, *K*, determines the maximum rank of the inferred covariance functions and is equivalent to the effective number of “traits” in the analysis. We tested models where *K* ranged from 2 to 8. Similar results were found for both *K *=* *7 and 8, with AIC favoring *K *=* *7 (see Supporting information Table S1 and Figure S1). Although it is possible that higher order polynomials would fit better, the models with *K *=* *7 and 8 already explain 91% of the variance in length. To avoid overfitting, we therefore restrict the analysis to 7 basis functions for the remainder of the paper. Further details of the model specification and the Gibbs sampler used to construct posteriors are in the Supporting Information, and the convergence plot is shown in Supporting information Figure S2.

Once the parameters were estimated**,** we used **C**_**s**_, **C**_**d**_, and **E** to construct the genetic and environmental covariance functions and used these to find the age‐specific heritability and maternal contribution. To go from the covariance matrix for the basis coefficients back to the corresponding covariance function, we apply the basis expansion to get $C\left(t,t^{\prime }\right)=\sum_{k=1}^{K}\sum_{l=1}^{K}h_{k}\left(t\right)h_{l}\left(t^{\prime }\right)C_{k,l}$.

To evaluate how representative a single estimate of heritability would be for the whole size trajectory, we calculated the heritability at age using the diagonal elements of the genetic and phenotypic covariance functions. Specifically, we calculated $h^{2}\left(t\right)=C_{s}\left(t,t\right)/C_{p}\left(t,t\right)$, where the phenotypic covariance function is given by $C_{p}\left(t,t^{\prime }\right)=\frac{3}{4}C_{s}\left(t,t^{\prime }\right)+\frac{1}{4}C_{d}\left(t,t^{\prime }\right)+C_{e}\left(t,t^{\prime }\right)$ (Lynch & Walsh, 1998). The maternal effects contribution are calculated as $m\left(t\right)=\frac{1}{4}\left[C_{d}\left(t,t\right)-C_{s}\left(t,t\right)\right]/C_{p}\left(t,t\right)$. Note that in the case where the genetic variances from the sire and dam are identical, we may write the heritability for size at age *t* in the more familiar way with $h^{2}\left(t\right)=V_{g}\left(t\right)/\left[V_{g}\left(t\right)+V_{\epsilon }\left(t\right)\right]$ with $V_{g}\left(t\right)=C_{s}\left(t,t\right)$ and $V_{\epsilon }\left(t\right)=C_{e}\left(t,t\right)$.

### 2.5 Response to size‐selective mortality

To illustrate the role of genetic covariances across ages and the potential utility of the function‐valued trait approach for fisheries‐induced evolution, we used the estimated genetic covariance functions to model the evolutionary response to size‐selective harvesting. For clarity, we simplify Equation (1) in the subsequent model by dropping the fixed effects and assuming that sire and dam genetic contributions have the same variance, so that

(2) $$y\left(t\right)=\mu \left(t\right)+g\left(t\right)+\epsilon \left(t\right)$$

The trajectory of fisheries‐induced evolution depends strongly on both gear selectivity (Andersen & Brander, 2009) and the size dependence of natural mortality (Jørgensen & Fiksen, 2010). Since our purpose here is to highlight the relevance of treating size as a FVT, we do not exhaustively evaluate the implications of different selectivity patterns. Rather we consider idealized versions of the two most commonly used gear selectivity patterns: sigmoid and unimodal (Kuparinen, Kuikka, & Merilä, 2009). For the sigmoid model, gear selectivity is modeled as $Q\left(y\right)={\left[1+\text{exp}\left(-s\left(y-\theta \right)\right)\right]}^{-1}$ where *y* is length, and the parameters θ and *s* govern the inflection point and slope at inflection, respectively. For the unimodal model, we used $Q\left(y\right)=\text{exp}\left[-{\left(y-\varphi \right)}^{2}/w\right]$ where φ and *w* govern the most catchable size and the width of the selectivity curve, respectively. In both cases, the maximum catchability is 1. We also assume that there is some size‐dependent natural mortality, which decays exponentially with length and is given by $N\left(y\right)=\text{exp}\left[-b\left(y-y_{0}\right)\right]$ where *y*_0_ is the size at hatch and b controls the rate at which natural mortality declines with size.

In the analyses presented here, we set θ to 95 mm and *s* to 0.15/mm for the sigmoid model, φ* *= 80 mm and *w *=* *150 mm^2^ for the unimodal model, and *y*_0_ = 7 mm and *b *=* *0.01/mm for the natural mortality model. Putting these together, total mortality at length *y* is given by $M\left(y\right)=mN\left(y\right)+fQ\left(y\right)$ where m and f scale the overall rates of natural and fishing mortality, respectively.

We assume that as a cohort ages, it experiences multiple rounds of selection from both natural and fishing mortality. However, to keep the model analytically tractable, we assume that fitness is determined by survival to maturation. Although size is a major determinant of fecundity in fishes (Barneche, Robertson, White, & Marshall, 2018), we do not have data on the covariance between fecundity and size at earlier ages and consequently choose to focus specifically on survival. This model characterizes the silverside life cycle fairly well prior to maturation and would be a reasonable approximation for other semelparous species. We note that this model does not apply to harvested populations with overlapping generations. The fitness calculation in such a case requires a more elaborate analysis, which will be the subject of a subsequent publication. Nevertheless, we believe that this model adequately illustrates how different assumptions on the genetic covariance can affect the predictions of models of fisheries‐induced evolution.

With these assumptions, the fitness of a given size trajectory, *y*(*t*) is found by integrating mortality from birth to reproduction, that is,

(3) $$W\left(y\right)=\text{exp}\left\{-\int_{0}^{T}M\left[y\left(t\right)\right]dt\right\}$$

We note that it would be relatively simple to include reproductive output, φ in this semelparous model, for example, $W\left(y\right)=\varphi \;\text{exp}\left\{-\int_{0}^{T}M\left[y\left(t\right)\right]dt\right\}$. However, fecundity is typically size dependent and likely to covary with size at several ages due to trade‐offs between growth and reproduction (Stearns, 1992). Since we do not have data on the genetic covariance between fecundity and length at age, we eschew making assumptions about this and restrict our attention to survival to maturation.

One approach to determining the evolutionary response is to evaluate Equation (3) by simulation which is particularly helpful for visualizing the fitness of different length trajectories. To do so, we simulated 1,000 length trajectories drawn from the Gaussian process in Equation (2) based on our estimates of the covariance functions. We then evaluated fitness for each trajectory directly using Equation (3). However, it is difficult to connect simulations to existing analytical results (e.g., the breeder's equation) or extract deeper insight into the effects of genetic covariances on fisheries‐induced evolution. To help clarify the role of genetic covariances, we approximate fitness with a quadratic form. This is the theoretical approach most often used in the multivariate evolution literature (see, e.g., Tufto 2017) and permits analytical calculation of the selection differential, gradient, and response to selection. To do so, we use a second‐order Taylor expansion of the mortality rate around the mean size at age, that is,

(4) $$W\left(y\right)∼\text{exp}\left\{-\int_{0}^{T}M\left[\mu \left(t\right)\right]+M^{\prime }\left[\mu \left(t\right)\right]\left[y\left(t\right)-\mu \left(t\right)\right]+\frac{1}{2}M^{\prime \prime }\left[\mu \left(t\right)\right]{\left[y\left(t\right)-\mu \left(t\right)\right]}^{2}dt\right\}$$

For sake of completeness, note that had we included fecundity in our fitness model and assumed that fecundity is solely a function of size at maturation, say $\varphi \left[y\left(T\right)\right]$, we would modify the quadratic form as $W_{f}\left(y\right)∼\text{exp}\left\{\ln\left\{\varphi \left[\mu \left(T\right)\right]\right\}+\left(\varphi^{\prime }/\varphi \right)\left[y\left(T\right)-\mu \left(T\right)\right]+\left(\varphi^{\prime \prime }/\varphi -{\left(\varphi^{\prime }/\varphi \right)}^{2}\right){\left[y\left(t\right)-\mu \left(t\right)\right]}^{2}\right\}W\left(y\right)$ where φ′ and φ*″* represent the first and second derivatives of fecundity evaluated at the mean length at time *T* and *W* (*y*) is the approximate fitness from Equation (4). Since fecundity is typically allometric in length, that is, *ϕ* = *cy* ^*d*^, $\frac{\varphi^{\prime }}{\varphi }=\frac{d}{y}$, and $\frac{\varphi^{\prime \prime }}{\varphi }-{\left(\frac{\varphi^{\prime }}{\varphi }\right)}^{2}=-\frac{d}{y^{2}}$. Moreover, the covariance between fecundity and length at age would then be driven by the covariance between terminal size and the length at earlier ages. However, this is a simplifying assumption that we do not pursue any further.

Using the basis expansion, $y\left(t\right)-\mu \left(t\right)=\sum_{k=1}^{K}h_{k}\left(t\right)\left(\gamma_{k}+\epsilon_{k}\right)$, Equation (4) can be rewritten as a quadratic form in terms of the basis coefficients. To simplify notation, let *z*_*k*_ be the sum of genetic and environmental components for the kth coefficient, that is, *z*_*k*_ = γ_*k*_ + ε_*k*_ Plugging this in to Equation (4), we get

(5) $$W\left(y\right)∼\text{exp}\left\{-\overset{¯}{M}-\sum_{k=1}^{K}m_{k}^{\left(1\right)}z_{k}-\frac{1}{2}\sum_{k=1}^{K}\sum_{l=1}^{K}z_{k}z_{l}m_{k,l}^{\left(2\right)}\right\}$$

where $\overset{¯}{M}=\int_{0}^{T}M\left(\mu \left(t\right)\right)\text{dt}$ is the total mortality for the mean size trajectory, $m_{k}^{\left(1\right)}=\int_{0}^{T}M^{\prime }\left(\mu \left(t\right)\right)h_{k}\left(t\right)\text{dt}$ is the projection of mortality gradient on the *k*th basis function, and $m_{k,l}^{\left(2\right)}=\int_{0}^{T}M^{\prime \prime }\left(\mu \left(t\right)\right)h_{k}\left(t\right)h_{l}\left(t\right)\text{dt}$ is the projection for the second derivative.

Recall that before selection *E* (*z*_*k*_) = 0 and the covariance matrix for ***z*** = {*z*_1_, …, *z*_*K*_}^*T*^ is given by ***P*** = ***C***_***s***_ + ***E*** (since we have assumed that C_s_ = C_d_). Moreover, since the basis coefficients are multivariate normal, we can use the standard formulae for multivariate evolution to find that the mean for ***z*** = {*z*_1_, …, *z*_*K*_}^*T*^ after selection is

(6) $${\overset{¯}{z}}^{*}=-{\left[P^{-1}+m^{\left(2\right)}\right]}^{-1}m^{\left(1\right)}$$

and the mean in the following generation is given by

(7) $$\Delta \overset{¯}{z}=C_{s}P^{-1}{\overset{¯}{Z}}^{*}$$

where ***m*** ^(**1**)^ is the vector of first‐derivative projections, that is, ***m*** ^(**1**)^ =${\left\{m_{1}^{\left(1\right)},\ldots ,m_{K}^{\left(1\right)}\right\}}^{T}$ ***m*** ^(**2**)^ and is the matrix of second‐derivative projections whose (*k*,*l*)^th^ element is $m_{k,l}^{\left(2\right)}$.

Inserting these results into the basis expansion, we find that the change in mean length at age t following selection is given by $\Delta {\overset{¯}{y}}^{*}\left(t\right)=\sum_{k=1}^{K}h_{k}\left(t\right){\overset{¯}{z}}_{k}^{*}$ and the evolutionary response, that is, the change in the population mean in the next generation, is given by $\Delta \overset{¯}{y}\left(t\right)=\sum_{k=1}^{K}h_{k}\left(t\right)\Delta {\overset{¯}{z}}_{k}$. We used these approximations to calculate the response to selection for both the sigmoid and unimodal gear selectivity. To examine the relative importance of fishing versus natural mortality, we repeated these calculations over a range of values for *m* and *f*.

### 2.6 Alternative assumptions about genetic covariances

In order to clarify the role of the genetic covariances in the response to selection, we consider two alternative models for the genetic basis of body size. In the first alternative model, we assume that size at age has constant heritability. As we show below, this is analogous using a single evolving trait to model fisheries‐induced evolution. The second alternative treats the size at each age as an independent character. Although we are unaware of analogues for this second assumption in the fisheries‐induced evolution literature, this model is the opposite extreme in terms of genetic constraints. Hence, the FVT approach based on the estimated genetic covariances is intermediate between these two alternatives.

For our first genetic alternative, heritability is constant across ages. But in order to make the results directly comparable with our baseline model, we constrain the phenotypic variance at age, *V*_*p*_ (*t*), to be the same as for our baseline model by setting $V_{g}\left(t\right)={\overset{¯}{h}}^{2}V_{p}\left(t\right)$, where ${\overset{¯}{h}}^{2}$ is the mean heritability over all ages. Note that this implies that the environmental variance is $V_{\epsilon }\left(t\right)=\left(1-{\overset{¯}{h}}^{2}\right)V_{p}\left(t\right)$ . To connect this with the general model, note that this is precisely the same as rewriting Equation (2) as

(8) $$y\left(t\right)=\mu \left(t\right)+\left(\tilde{g}+\tilde{\epsilon }\right){\left[V_{p}\left(t\right)\right]}^{1/2}$$

where $\tilde{g}$ and $\tilde{\epsilon }$ are constants representing the genetic and environmental components, scaled such that $V\left(\tilde{g}\right)={\overset{¯}{h}}^{2}$ Moreover, this is equivalent to setting *K *=* *1 and using single basis function given by $h_{1}\left(t\right)={\left[V_{p}\left(t\right)\right]}^{1/2}$. Since *K* is analogous to the number of traits in the analysis, we refer to this as the “single‐trait” model.

The genetic and environmental covariance functions for the single‐trait model are ${C_{s}}^{\mathrm{one}}\left(t,t^{\prime }\right)=V\left(\tilde{g}\right){\left[V_{p}\left(t\right)V_{p}\left(t^{\prime }\right)\right]}^{1/2}$ and ${C_{\epsilon }}^{\mathrm{one}}\left(t,t^{\prime }\right)=V\left(\tilde{\epsilon }\right){\left[V_{p}\left(t\right)V_{p}\left(t^{\prime }\right)\right]}^{1/2}$. This implies the correlation (both genetic and environmental) between size at any pair of ages is 1. Moreover, since there is only one component in this model, Equation (6) and (7) reduce to: ${\overset{¯}{Z}}^{*}=-m^{\left(1\right)}/\left[1+m^{\left(2\right)}\right]$ and $\Delta \overset{¯}{z}={\overset{¯}{h}}^{2}{\overset{¯}{z}}^{*}$ where $m^{\left(1\right)}=\int_{0}^{T}M^{\prime }\left(\mu \left(t\right)\right){\left[V_{p}\left(t\right)\right]}^{1/2}\text{dt}$ and $m^{\left(2\right)}=\int_{0}^{T}M^{\prime \prime }\left(\mu \left(t\right)\right){\left[V_{p}\left(t\right)\right]}^{1/2}\text{dt}$. Hence, the response to selection in the single‐trait model is determined by a weighted average of the selection applied to each age.

At first glance, the single‐trait model might seem like an unreasonably oversimplified abstraction. To put it into a more familiar context, consider what happens if we assume von Bertalanffy growth in length, $\frac{\text{dL}}{\text{dt}}=a-bL$, where *a* represents anabolic processes and *bL* represents catabolic processes (e.g., Vincenzi, Mangel, Crivelli, Munch, & Skaug, 2014). Assume that there is genetic variation in the anabolic term such that $a=\overset{¯}{a}+a_{g}+a_{\varepsilon }$. Integrating from an initial size of 0, we get *L*(*t*) = *a* (1 − e^‐*bt*^)/*b*. Decomposing *a* into genetic and environmental components and making the analogy with Equation (3), we have $\mu \left(t\right)=\overset{¯}{a}\left(1-e^{-bt}\right)/b$, $g\left(t\right)=a_{g}\left(1-e^{-bt}\right)/b$, and $\epsilon \left(t\right)=a_{\epsilon }\left(1-e^{-bt}\right)/b$, from which we can derive the covariance and other functions. For example, the genetic covariance in size at age is $E\left[g\left(t\right)g\left(t^{\prime }\right)\right]=\left[\text{Var}\left(a_{g}\right)+\text{Var}\left(a_{\epsilon }\right)\right]\left(1-e^{-bt}\right)\left(1-e^{-bt^{\prime }}\right)/b^{2}$ and the variance in length at age *t* is $V_{p}\left(t\right)=\left[\text{Var}\left(a_{g}\right)+\text{Var}\left(a_{\epsilon }\right)\right]{\left(1-e^{-bt}\right)}^{2}/b^{2}$. Putting these together, the heritability for size at age is constant at $h^{2}=\text{Var}\left(a_{g}\right)/\left[\text{Var}\left(a_{g}\right)+\text{Var}\left(a_{\epsilon }\right)\right]$. From this, we can see that under some circumstances, assuming genetic variation in a single parameter is the same as using Equation (8). Note that things are not always this simple; assuming genetic variation in *b* would also have resulted in a rank 1 covariance function, but without the tidy additive decomposition in Equation (1), (2), and (8).

For our second alternative genetic model, we assume that the size at each age is an independent trait. Although this is physiologically impossible, it is the polar opposite of the single trait alternative in terms of evolutionary flexibility. Under this model, we keep the genetic and environmental variances the same as in our baseline model, but force the covariances to be 0. Under this model, the age‐specific heritabilities are the same as for the baseline model and evolution of size at each age follows a univariate breeders equation, that is, $\Delta \overset{¯}{y}\left(t\right)=h^{2}\left(t\right)\Delta {\overset{¯}{y}}^{*}\left(t\right)$.

## 3 RESULTS

**Figure 1:**
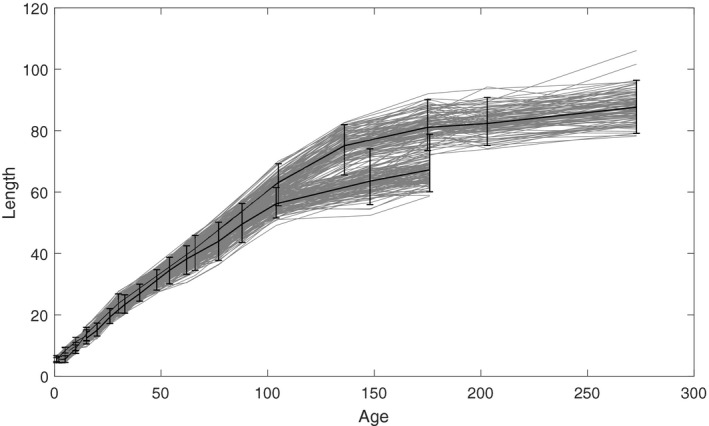
Growth trajectories over the entire lifespan. The three rounds of experiments are plotted together. The error bars indicate 95% confidence intervals. The first batch only lasted for 15 days posthatch and is overlapping with the other two batches at the beginning of the trajectories

**Figure 2:**
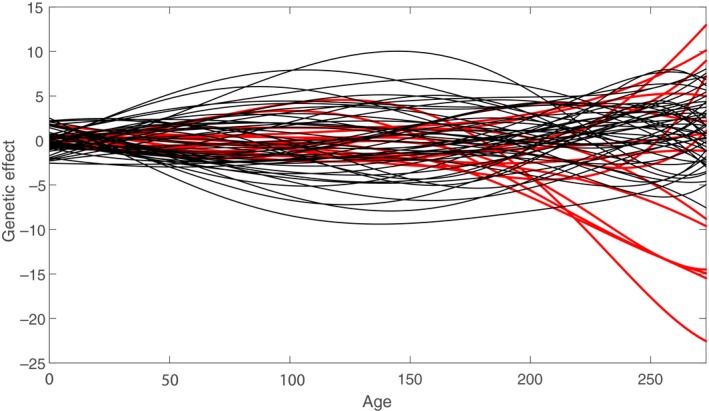
The estimated genetic effects for individual sire (red) and dam (black)

**Figure 3:**
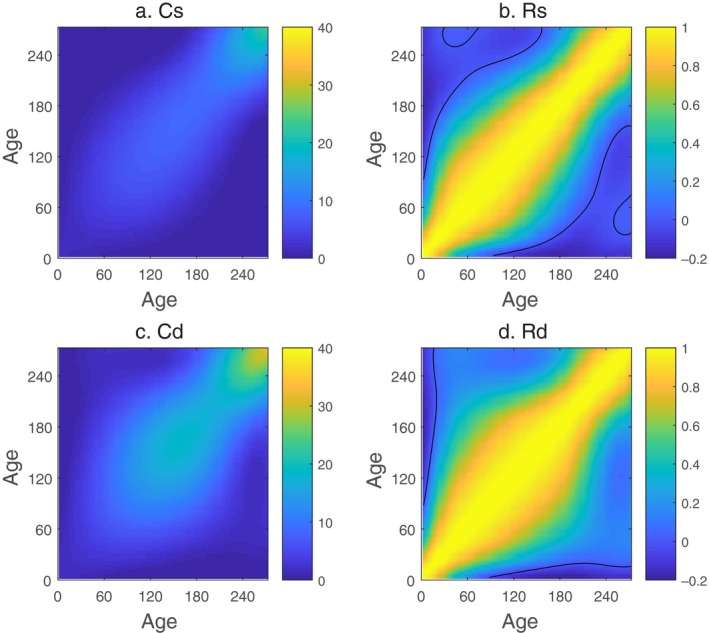
Contour plots of the estimated genetic variance–covariance function and the corresponding correlation functions. The variance–covariance function and corresponding correlation functions for sire covariance functions are shown in (a) and (b). The variance–covariance function and corresponding correlation functions for dam effects are shown in (c) and (d). Zero contour line is shown in black

The growth trajectories from each of the three experimental batches are presented in Figure 1. The growth trajectories are asymptotic as is typically observed in fish. The estimated sire and dam contributions tend to cluster closely together in early ages while the variance among them increases with time (Figure 2). The inferred sire covariance function is somewhat smaller than the dam covariance overall, suggesting the presence of maternal effects (Figure 3 *C*_*s*_ and *C*_*d*_).

The genetic correlation among ages varies through time. Although it is the genetic covariance that determines the rate and path of evolution in response to a given form of selection, changes in scale may make it difficult to think about. For instance, if length at age was given by *L *= *bt*, the covariance in length 2 days apart would be Var(b)[*t* ^2^ +2*t*]. This covariance clearly increases with age obscuring the fact that the lengths at all ages are perfectly correlated. The genetic correlation function circumvents this by scaling out the change in variance through time, for example, $R_{s}\left(t,t^{\prime }\right)=C_{s}\left(t,t^{\prime }\right)/{\left[C_{s}\left(t,t\right)C_{s}\left(t^{\prime },t^{\prime }\right)\right]}^{1/2}$ (Figure 3). The pattern in *R*_*s*_ indicates that size at similar ages are more correlated than size at ages that are far apart. There is a weak negative correlation between lengths at early ages and ages ~150–200 days. This is less pronounced in the correlations estimated from dam effects (Figure 3d), suggesting mitigation through maternal contributions.

**Figure 4:**
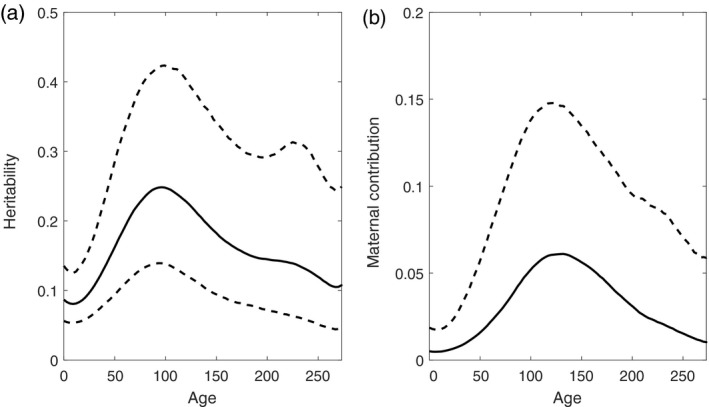
Heritability estimates (a) and maternal contributions (b) over the entire duration. The solid line is the average heritability over time, and the upper and lower dashed lines indicate the 95% credibility interval. The lower interval of maternal contributions remains zero

The genetic and maternal contributions to length varied with offspring age. Heritability throughout the lifespan is plotted in Figure 4a. Heritability was small (0.08) at hatch and peaked at 0.25 on day 100. Following day 100, heritability decreased steadily. The heritability at day 200 is roughly 0.15 (with 95% confidence interval from 0.07 to 0.29), which is generally consistent with previous finding that the realized heritability was ~0.2 at age 190 days (Conover & Munch, 2002). The maternal contribution was overall quite low. The estimated maternal contribution to initial size was 0.005, which increased to 0.088 at 135 days and then declined (Figure 4b).

### 3.1 Response to selection

**Figure 5:**
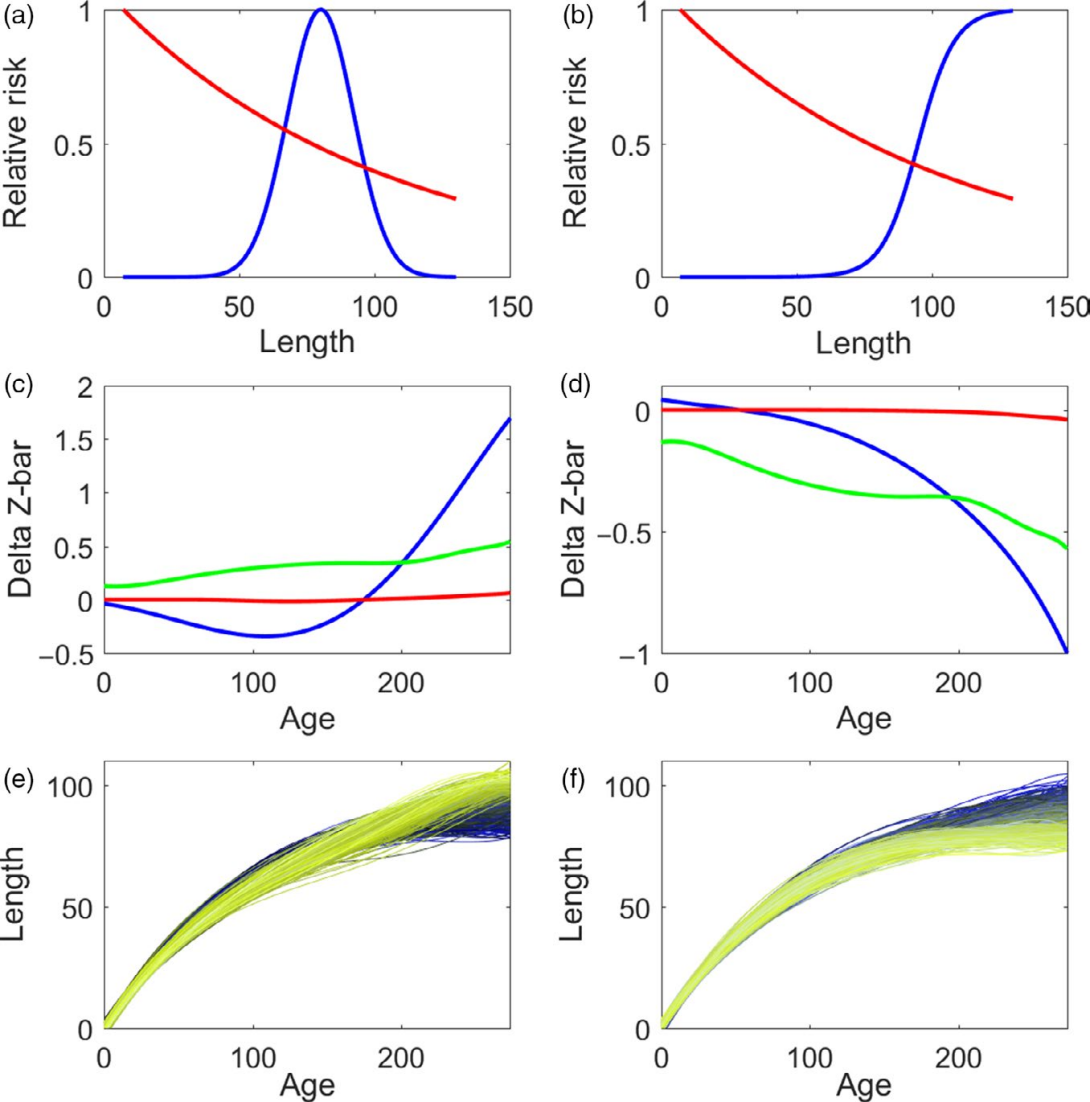
Simulation results using estimated genetic variance–covariance functions and hypothetical gear selectivity curves. The a, c, and e panels show the unimodal selectivity curve (a), the predicted changes in mean size over one generation under full covariance function (blue), constant heritability (green) and independent traits (red) (c), and simulated phenotypic changes (e) under unimodal selectivity. The b, d, and f panels show the sigmoid selectivity curve (b), the predicted changes in mean size over one generation under full covariance function (blue), constant heritability (green) and independent traits (red) (d), and simulated phenotypic changes (f) under unimodal selectivity. The fishing mortality used is 0.0545, and natural mortality used is 0.0109

Our hypothetical sigmoid and unimodal selectivity curves generated qualitatively different responses to selection. Under sigmoid selectivity, simulated length trajectories with the greatest fitness are those that stop growing by 80 mm, at which point the fishing mortality is still less than 20% of its maximum (Figure 5b,f). In contrast, our unimodal selectivity example favored length trajectories that grew slower than average prior to 50 mm and rapidly thereafter, ending up larger than average by day 270 (Figure 5e). To see why this was so, we can change variables in (3) to get $\text{ln}\left[W\left(y\right)\right]=-\int_{y\left(0\right)}^{y\left(T\right)}M\left[y\right]\tau \left(y\right)\text{dy}$ where τ(*y*) = [dy/dt]^−1^ is the time spent at size *y*. Hence, all else being equal, we expect evolution to favor growth trajectories that minimize the time over which an individual is exposed to harvesting.

The predicted evolutionary responses to a single generation of selection under these two gear selectivity patterns depend heavily on the genetic covariance between size at different ages. Under our sigmoid selectivity scenario and the full genetic covariance model, offspring length is predicted to increase for the youngest fish up to about 40 days and decrease at all ages thereafter in a roughly parabolic manner (Figure 5d), consistent with the fitness for the simulated trajectories. The single‐trait model misses the increase in size in young fish, predicting that size will decrease at all ages. Compared to the full covariance, the single‐trait model overestimates the magnitude of the decrease up to about 100 days and underestimates it thereafter. The response to selection for the independent traits model is negligible except for the oldest ages.

Under our unimodal selectivity scenario and the full genetic covariance function, the predicted response is somewhat more complicated (Figure 5c). Offspring length is predicted to decrease in fish less than ~180 days with a maximum decline of about 0.25 mm in length at age ~100 days. For fish older than ~180 days length is predicted to increase. Again, this prediction is consistent with the fitness of simulated trajectories. In contrast, the single‐trait model predicts that size should increase at all ages, more for older fish. The response to selection for the independent traits model is negligible except for the oldest ages.

**Figure 6:**
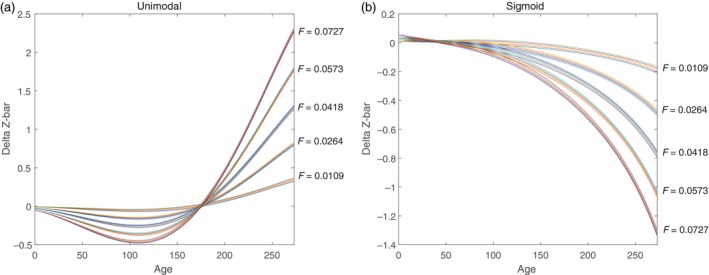
Simulation results using the complete combination of fishing mortality of (0.0109, 0.0264, 0.0418, 0.0573, 0.0727) marked on the figure. Each *F* has five separate simulations using natural mortalities of (0.0018, 0.0059, 0.01, 0.0141, 0.0182)

We repeated these calculations for a range of values for *m* and *F* to evaluate the relative importance of natural and harvest selection (Fig. 6). In the scenarios we tested, the predicted responses to selection are much more sensitive to changes in *F* than *m* for both the sigmoid and unimodal gear selectivity patterns.

## 4 DISCUSSION

### 4.1 Genetic covariance function

Both parametric and nonparametric methods have been developed to estimate the covariance functions, such as least‐square estimates (Kirkpatrick & Heckman, 1989; Kirkpatrick, Hill, & Thompson, 1994), restricted maximum likelihood (Meyer & Hill, 1997), and random regression (Meyer, 1998). Several advantages exist when combining the function‐valued trait approach with a Bayesian hierarchical model. The method used in this study guarantees the positive definiteness of the covariance matrices and easily provides credibility intervals. It does not require a balanced design or fixed measurement intervals and also provides a straightforward path to combining multiple datasets.

The genetic variance in length increased with age in both additive and maternal estimates. Since fish increase in size with age, the net effect of both genetic and nongenetic factors on growth accumulates through time, leading to this increasing trend in variance (Figure 2). The pattern for heritability was somewhat more complicated, with low values at both early and late ages and a peak of about 0.3 at 100 days. This variation in heritability with age is consistent with observations in other species (e.g., McKay et al., 1986; Wilson, Hutchings, & Ferguson, 2003) and suggests that a single heritability estimate can be quite misleading.

Size at age in silversides tends to be highly autocorrelated over intervals of 30 days or more. In particular, we found evidence of strong positive genetic correlations, particularly among ages 30–150 days. Interestingly, size early in life was negatively correlated with size over 150–200 days, indicating that fast‐growing juveniles tended to become smaller than average adults. These results are consistent with those in other species. In Soay sheep (*Ovis aries*), genetic correlations among ages were estimated for free‐living individuals from birth to 5 years old and positive covariances were found throughout ontogeny (Wilson et al., 2007). Similarly, in juvenile brook charr (*Salvelinus fontinalis*), significant positive genetic correlations for length at age were found (Wilson et al., 2003). Together, these findings suggest great evolutionary potential in response to intensive selection, albeit in potentially constrained directions.

### 4.2 Implications for fisheries‐induced evolution

In order to determine the importance of genetic correlations to modeling fisheries‐induced evolution, we estimated the response to a single generation of harvest selection under sigmoid and unimodal gear selectivity patterns in this semelparous fish. Although the specific magnitude of effects obviously depends on the choice of *m*,* F*, and the shape parameters of gear selectivity, several important observations emerged from these numerical experiments. The first is that the assumed shape of the gear selectivity function is important in shaping the response. In this model, a unimodal pattern of selectivity strongly favors fish that can grow rapidly through the window of high catchability. This is consistent with the theoretical predictions on evolution under slot limits (Dieckmann et al., 2006). In contrast, under sigmoid selectivity, we found that juvenile growth is relatively unaffected but that maximum size is likely to be sharply reduced.

The second observation to emerge from this exercise is that under both selectivity scenarios results were much more sensitive to changes in fishing mortality than to changes in natural mortality. This result depends both on the amount of genetic variation for size at age estimated in the experiment and the cumulative exposure to each source of mortality assumed in the selection model. Growth is fastest during the early life history, so individuals tend to rapidly outgrow the interval of high natural mortality. In contrast, slow growth among older fish prolonged their exposure to harvest selection, particularly under sigmoid selectivity. Hence, even if *m* and *F* were the same, the cumulative impact of fishing would be greater. Although this model is fairly contrived, we expect that, all else being equal, these intuitively reasonable results should be reasonably general.

The final observation from these calculations is that genetic correlations have a substantial impact on the outcome. The “single‐trait” model, analogous to allowing a single growth parameter to evolve, represents the strongest constraints on the possible paths of evolution. Using this model, selection is averaged over the entire life history. Consequently, size across all ages can only increase (decrease) if the net selection is positive (negative). This is clearly different than what was observed with the full covariance function under unimodal selectivity, where the shape of the growth trajectory changed such that length at some ages increased while others decreased. In light of this, we suggest that future simulations of fisheries‐induced evolution would benefit from a more flexible representation for the genetic basis of growth.

In contrast, treating length at each age as a sequence of independent traits virtually eliminated any response to selection in this modeling exercise. Given previous theory on how genetic correlations can constrain evolution (Roff, 1996; Lynch & Walsh, 1998), this was initially somewhat counterintuitive. However, the change mean length at age is the sum (integral) of both the direct effects of selection at that age and the indirect effects of selection on correlated ages. When selection acts in the same direction over all correlated ages, the net effect will, in general, be considerably greater than the direct effect alone. Among older fish in our model, size at age is very strongly correlated for ~60 days (30 days in either direction) over which selection is always in the same direction. In retrospect, we should have expected the response using the full covariance function to be on the order of 60 ×  greater than the response in the independent traits model.

Compared to most of the commercial species with overlapping generations and size‐dependent fecundity, our model is oversimplified. However, it does illustrate that the response to selection on body size depends heavily on the particular ages over which selection acts and that this is driven by a combination of the size specificity of selection and the time spent at a given size. This is particularly relevant since rapid evolution of harvested populations has been widely documented (Haugen & Vøllestad, 2001; Conover & Munch, 2002; Law, 2007; Enberg et al., 2011) and tends to focus on the largest (and slowest growing) individuals in a given cohort.

### 4.3 Caveats on the function‐valued trait approach

Although not our main focus, we close with some caveats regarding implementation and interpretation of FVT analyses. Our intention here is to save future practitioners from the making same time‐consuming mis‐steps we did. In keeping with most recent literature on function‐valued traits, we used a basis function expansion to approximate the trait shape. Although all reasonable basis sets are complete in the limit as *K* → ∞ (i.e., can take on almost any shape), statistical approaches invariably truncate the expansion to some relatively small number of functions. As noted by Griswold et al. (2008), the efficiency and suitability of a given basis set depend heavily on the nature of the data. To avoid the problem of overfitting, Griswold et al. (2008) suggested thoroughly comparing the efficiencies of the different basis sets (defined as the number of basis functions needed to achieve a given level of approximation accuracy). This is a sound recommendation that we reiterate.

Griswold et al. (2008) found that the cosine basis was the most efficient for the growth trajectories they analyzed. Though clearly not a problem in their analysis, we found that some care must be taken to avoid introducing artifacts to the inferred covariance functions. We had initially used $h_{k}\left(t\right)=\text{cos}\left(\pi kt\right)$ as a basis with *k *=* *0,1, … *K* and found that we could match the growth trajectories reasonably well with *K *=* *4. However, the variance at time *t* is given by $V\left(t\right)=\sum_{k=1}^{4}\sigma_{k}^{2}{\text{cos}}^{2}\left(\pi kt\right)$, which is clearly a periodic function. It is an open question whether the resulting pattern of local minima and maxima correspond to anything biological. It seems more likely to us that the resulting peaks and troughs in variance are artifacts of using a truncated basis.

Importantly, similar artifacts can arise in any *truncated* basis expansion. To illustrate this, we plot the inferred genetic correlation using Legendre polynomials with *K* ranging from 2 to 8 (Supporting information Figure S1). With only two basis functions, length is perfectly correlated for all ages greater than 100 days. This changes markedly when we move to four basis functions and the long‐range correlations vanish. As we move from 4 to 8 basis functions the range of ages that are highly correlated narrows progressively.

In addition, we note that fixing K places a hard upper bound on the rank of the estimated genetic covariance function. This is the same thing as determining *a priori* the number of “traits.” As our model for the response to selection demonstrates, setting K too small may artificially limit the predicted paths that evolution may take. An alternative approach to using model selection to determine the number of basis functions would be to make K fairly large and use a prior (or penalty function, depending on your statistical persuasion) that shrinks coefficients toward zero. In this way, we might avoid potential biases in the evolutionary inferences drawn using the function‐valued trait approach.

## CONFLICT OF INTEREST

None declared.

## DATA ARCHIVING STATEMENT

Data available from the Dryad Digital Repository: https://doi.org/10.5061/dryad.tq2f566.
