# Supplementary material for: A function‐valued trait approach to estimating the genetic basis of size at age and its potential role in fisheries‐induced evolution
Source: Evol Appl. 2019 Feb 27;12(5):964–76. doi: 10.1111/eva.12771 (PMC6503830; doi:10.1111/eva.12771)

**Supporting Information**

Following Eq 1 in the main text, the function valued trait model for an individual length trajectory, y_i_(t) is defined as

$y_{i}\left( t \right)=\mu\left( t \right)+\beta_{i}\left( t \right)+\sum_{k=1}^{K} h_{k}\left( t \right)\left[ \gamma_{s,k}+\gamma_{d,k} \right]/2+\varepsilon_{i}\left( t \right)$ (Eq. S1)

For measurements at times {t_1_,…,t_m_}, the lengths for individual i can be stacked in a vector **y**_i_ = {y_i_(t_1_),…,y_i_(t_m_)}^T^

$\mathbf{y}_{\mathbf{i}}=\boldsymbol{\mu}+\boldsymbol{\beta}_{i}+\mathbf{H}\left[ \boldsymbol{\gamma}_{s}+\boldsymbol{\gamma}_{d} \right]/2+\boldsymbol{\varepsilon}_{i}$ (Eq. S2)

Where $\boldsymbol{\mu}$**,** $\boldsymbol{\beta}_{i}$, and $\boldsymbol{\varepsilon}_{i}$ are m x 1 vectors the mean size across all families, mean sizes for the family and environmental errors for the family it comes from respectively. **H** is an m x K basis expansion matrix and $\boldsymbol{\gamma}_{s}\mathrm{and}\boldsymbol{\gamma}_{d}$ are K x 1 vectors for basis expansion coefficients. The environmental errors, $\boldsymbol{\varepsilon}_{i}$, are zero-mean multivariate normal random variables with covariance matrix$\mathbf{E}$. Although we have observations on all individuals alive at each time point, our tags permit identification only to family. As a consequence, we work with the family means (identified by sire s and dam d)

${\bar{\mathbf{y}}}_{\mathrm{sd}}=\boldsymbol{\mu}+\boldsymbol{\beta}_{\mathrm{sd}}+\mathbf{H}\left[ \boldsymbol{\gamma}_{s}+\boldsymbol{\gamma}_{d} \right]/2+{\bar{\boldsymbol{\varepsilon}}}_{\mathrm{sd}}$ (Eq. S3)

From standard normal theory, the environmental error vector ${\bar{\boldsymbol{\varepsilon}}}_{\mathrm{sd}}\sim N(\mathbf{0},\bar{\mathbf{E}})$ where ${\bar{\mathbf{E}}}_{\mathrm{sd}}=\mathbf{E}\frac{1}{n_{\mathrm{sd}}}$ if we observe $n_{\mathrm{sd}}$ individuals. Unfortunately, not all individuals survive to be measured at all time points. In this case the covariance is somewhat more complicated, ${\bar{\mathbf{E}}}_{\mathrm{sd}}=\mathbf{N}_{\mathrm{sd}}^{-1/2}\mathbf{E}\mathbf{N}_{\mathrm{sd}}^{-1/2}$ where $\mathbf{N}_{\mathrm{sd}}$is the matrix obtained by putting $\bar{n}_{\mathrm{sd}}$, the average of $\{n_{\mathrm{sd}}\left( t_{1} \right),\ldots, n_{\mathrm{sd}}(t_{m})\}$ on the diagonal. Thus, conditional on all fixed and genetic effects, the likelihood for the family mean ${\bar{\mathbf{y}}}_{\mathbf{sd}}$ is multivariate normal with mean $\mathbf{m}_{\mathbf{sd}}\boldsymbol{=\mu}+\boldsymbol{\beta}_{\mathrm{sd}}+\mathbf{H}\left[ \boldsymbol{\gamma}_{s}+\boldsymbol{\gamma}_{d} \right]/2$ and covariance ${\bar{\mathbf{E}}}_{\mathbf{sd}}$. However, we also have additional data on the variance within families at each time point, $\mathbf{s}_{\mathrm{sd}}^{2}\boldsymbol{=\{}s_{\mathrm{sd}}^{2}\left( t_{1} \right),\ldots,s_{\mathrm{sd}}^{2}\left( t_{m} \right)\}$**.** Including these in the likelihood for each family, we get

$\mathbf{p(}{\bar{\mathbf{y}}}_{\mathbf{sd}}\mathbf{,}\mathbf{s}_{\mathrm{sd}}^{2}\left| \boldsymbol{\mu},\boldsymbol{\beta}_{\mathrm{sd}},\boldsymbol{\gamma}_{s},\boldsymbol{\gamma}_{d},\mathbf{E} \right)$=

$\frac{1}{\sqrt{2\pi}}{|{\bar{\mathbf{E}}}_{\mathrm{sd}}|}^{-1/2}\exp\{-\frac{1}{2}\left( {\bar{\mathbf{y}}}_{\mathbf{sd}}-\mathbf{m}_{\mathbf{sd}} \right)^{T}{{\bar{\mathbf{E}}}_{\mathrm{sd}}}^{-1}\left( {\bar{\mathbf{y}}}_{\mathbf{sd}}-\mathbf{m}_{\mathbf{sd}} \right)-\frac{1}{2}\sum_{j=1}^{m} s_{\mathrm{sd}}^{2}\left( t_{j} \right)\left[ n_{\mathrm{sd}}\left( t_{j} \right)-1 \right]/\mathbf{E}_{\mathrm{jj}}\}$

(Eq. S4)

Where $\mathbf{E}_{\mathrm{jj}}$ denotes the diagonal elements of $\mathbf{E}$.

To put together the data for all N_f_ families (i.e. **Y**={$\bar{\mathbf{y}}$**_11_**^T^,…,${{\bar{\mathbf{y}}}_{\mathrm{Nf}}}^{T}$}^T^), it is convenient to stack the fixed effects as well as the sire and dam basis coefficients yet again. These are denoted **b (**N_f_m x 1**)**, **g_s_** (N_s_K x 1) and **g_d_** (N_d_K x 1) for all N_s_ sires and N_d_ dams respectively. Let **X**, **S**, and **D** be the corresponding indicator matrices for the fixed effects, sires, and dams. If $\mathbf{ℇ}$ (N_f_m x 1) summarizes the environmental effects in a vector, the linear model for the full data set can then be written as

$\mathbf{Y}=\mathbf{(I}_{\mathbf{n}}\boldsymbol{\bigotimes\mu)}+\mathbf{Xb}+\mathbf{(I}_{\mathbf{n}}\boldsymbol{\bigotimes H)}\left[ \left( \boldsymbol{S\bigotimes}\mathbf{I}_{\mathbf{p}} \right)\mathbf{g}_{s}+({\boldsymbol{D\bigotimes}\mathbf{I}_{\mathbf{p}}\mathbf{)g}}_{d} \right]/2+\mathbf{ℇ}$ (Eq. S5)

Where **I_q_** is a q x q identity matrix and $\boldsymbol{\bigotimes}$ denotes the Kronecker product. The likelihood for **Y** is thus multivariate normal.

*S1. Gibbs sampler*

Let $\boldsymbol{\Sigma}$ be the block diagonal matrix with each block equal to ${\bar{\mathbf{E}}}_{\mathrm{sd}}$. If we also combine $\mathbf{(I}_{Nf}\boldsymbol{\bigotimes X)}$ into $\mathbf{X}^{\boldsymbol{*}}$ where the first column of $\mathbf{X}^{\boldsymbol{*}}$ being **1** and $\mathbf{(I}_{Nf}\boldsymbol{\bigotimes H)}$***(**$\boldsymbol{S\bigotimes}\mathbf{I}_{K}\boldsymbol{)}$ into $\mathbf{S}^{\boldsymbol{*}}$ and $\mathbf{(I}_{Nf}\boldsymbol{\bigotimes H)}$***(**$\boldsymbol{D\bigotimes}\mathbf{I}_{K}\boldsymbol{)}$ into $\mathbf{D}^{\mathbf{*}}$, the overall likelihood for data becomes Eq. S6, in which **R** is the sample variance matrix for each family with diagonal elements equal to $s_{\mathrm{sd}}^{2}\left( t_{j} \right)\left[ n_{\mathrm{sd}}\left( t_{j} \right)-1 \right]$ for j from 1 to m. $\boldsymbol{\Sigma}_{\boldsymbol{ii}}$ denotes the diagonal elements of $\boldsymbol{\Sigma}$.

${P\left( \mathbf{Y, R} | \boldsymbol{\Sigma}, \mathbf{b,}\mathbf{g}_{\mathbf{s}}\mathbf{,}\mathbf{g}_{\mathbf{d}} \right)\boldsymbol{\propto}\left| \boldsymbol{\Sigma} \right|}^{\mathbf{-}\frac{\mathbf{1}}{\mathbf{2}}}\exp\left\{ -\frac{1}{2}\left( \mathbf{Y-}\mathbf{X}^{\boldsymbol{*}}\mathbf{b-}\frac{1}{2}\mathbf{S}^{\boldsymbol{*}}\mathbf{g}_{\mathbf{s}}\mathbf{-}\frac{1}{2}\mathbf{D}^{\boldsymbol{*}}\mathbf{g}_{\mathbf{d}} \right)^{\mathbf{T}}\boldsymbol{\Sigma}^{-1}\left( \mathbf{Y-}\mathbf{X}^{\boldsymbol{*}}\mathbf{b-}\frac{1}{2}\mathbf{S}^{\boldsymbol{*}}\mathbf{g}_{\mathbf{s}}\mathbf{-}\frac{1}{2}\mathbf{D}^{\boldsymbol{*}}\mathbf{g}_{\mathbf{d}} \right)-\frac{1}{2}\boldsymbol{R\cdot}\boldsymbol{\Sigma}_{\boldsymbol{ii}}^{\boldsymbol{-1}} \right\}$ (Eq. S6)

The priors and hyper priors in the Gibbs sampler are listed below.

$${P\boldsymbol{(\gamma}}_{\mathbf{s}}\mathbf{|}\mathbf{C}_{\mathbf{s}}\boldsymbol{)\sim}\mathrm{MVN}\mathbf{(0,}\mathbf{C}_{\mathbf{s}}\boldsymbol{))\propto}\left| \mathbf{C}_{\mathbf{s}} \right|^{\mathbf{-}\frac{\mathbf{s}}{\mathbf{2}}}exp(-\frac{1}{2}{\boldsymbol{\gamma}_{\mathbf{s}}}^{T}{\mathbf{C}_{\mathbf{s}}}^{\mathbf{-1}}\boldsymbol{\gamma}_{\mathbf{s}}\mathbf{)}$$

$P\boldsymbol{(\gamma}_{\mathbf{d}}\mathbf{|}\mathbf{C}_{\mathbf{d}}\boldsymbol{)\sim}\mathrm{MVN}\mathbf{(0,}\mathbf{C}_{\mathbf{d}}\boldsymbol{)\propto}\left| \mathbf{C}_{\mathbf{d}} \right|^{\mathbf{-}\frac{\mathbf{d}}{\mathbf{2}}}exp(-\frac{1}{2}{\boldsymbol{\gamma}_{\mathbf{d}}}^{T}{\mathbf{C}_{\mathbf{d}}}^{\mathbf{-1}}\boldsymbol{\gamma}_{\mathbf{d}})$

P$\left( \mathbf{b} | V_{b} \right)\boldsymbol{\sim}\mathrm{MVN}\left( \mathbf{0,}V_{b} \right)\boldsymbol{\propto}\left| V_{\mathbf{b}}\mathbf{I}_{\mathbf{n}} \right|^{\mathbf{-}\frac{\mathbf{1}}{\mathbf{2}}}exp(-\frac{1}{2}b^{T}{{(V}_{\mathbf{b}}\mathbf{I}_{\mathbf{n}}\boldsymbol{)}}^{\mathbf{-1}}b\mathbf{)}$, in which $V_{b}=10000$.

$\mathbf{C}_{\mathbf{s}}\boldsymbol{\sim}\mathrm{Wishart}^{-1}\left( \boldsymbol{\Lambda,}s \right)$, in which $\boldsymbol{\Lambda}$ is an identity matrix of size K**.**

$\mathbf{C}_{\mathbf{d}}\boldsymbol{\sim}\mathrm{Wishart}^{-1}\left( \boldsymbol{\Lambda,}d \right)$, in which $\boldsymbol{\Lambda}$ is an identity matrix of size K**.**

$\boldsymbol{\Sigma\sim}\mathrm{Wishart}^{-1}\left( \boldsymbol{\Lambda}_{\mathbf{e}}\mathbf{,}m \right)$, in which $\boldsymbol{\Lambda}_{\mathbf{e}}$ is an identity matrix of size m**.**

A Gibbs sampler was used to estimate all the hyper parameters. The full conditional distributions of the parameters and the hyper parameter are listed.

$P\left( \mathbf{C}_{\mathbf{s}} | \cdot\right)\propto\mathrm{Wishart}^{-1}(s,\mathbf{g}_{\mathbf{s}}{\mathbf{g}_{\mathbf{s}}}^{T}+\boldsymbol{\Lambda})$

$P\left( \mathbf{C}_{\mathbf{d}} | \cdot\right)\propto\mathrm{Wishart}^{-1}(d,\mathbf{g}_{\mathbf{d}}{\mathbf{g}_{\mathbf{d}}}^{T}+\boldsymbol{\Lambda})$

$P\left( \boldsymbol{\Sigma} | \boldsymbol{\cdot} \right)\sim\mathrm{Wishart}^{-1}(N_{f},\left( \mathbf{Y-}\mathbf{X}^{\boldsymbol{*}}\mathbf{b-}\frac{1}{2}\mathbf{S}^{\boldsymbol{*}}\mathbf{g}_{\mathbf{s}}\mathbf{-}\frac{1}{2}\mathbf{D}^{\boldsymbol{*}}\mathbf{g}_{\mathbf{d}} \right)^{\mathbf{T}}\left( \mathbf{Y-}\mathbf{X}^{\boldsymbol{*}}\mathbf{b-}\frac{1}{2}\mathbf{S}^{\boldsymbol{*}}\mathbf{g}_{\mathbf{s}}\mathbf{-}\frac{1}{2}\mathbf{D}^{\boldsymbol{*}}\mathbf{g}_{\mathbf{d}} \right)+\boldsymbol{\Lambda}_{\mathbf{e}}+\mathbf{R})$

$P\left( \mathbf{g}_{\mathbf{s}} | \cdot\right)=MVN(\boldsymbol{\mu}_{\mathbf{s}}\mathbf{,}\boldsymbol{\Sigma}_{\mathbf{gs}})$

$$\boldsymbol{\Sigma}_{\mathbf{gs}}={(\frac{1}{4}{\mathbf{S}^{\mathbf{*}}}^{\mathbf{T}}\left( \boldsymbol{\Sigma} \right)^{\mathbf{-1}}\mathbf{S}^{\mathbf{*}}\mathbf{+}{\mathbf{C}_{\mathbf{s}}}^{\mathbf{-1}})}^{-1}$$

$$\boldsymbol{\mu}_{\mathbf{s}}=\frac{1}{2}\boldsymbol{\Sigma}_{\mathbf{gs}}{\mathbf{S}^{\mathbf{*}}}^{\mathbf{T}}\left( \boldsymbol{\Sigma} \right)^{\mathbf{-1}}\mathbf{(y-}\mathbf{X}^{\boldsymbol{*}}\mathbf{b-}\frac{1}{2}\mathbf{D}^{\boldsymbol{*}}\mathbf{g}_{\mathbf{d}}\mathbf{)}$$

$P\left( \mathbf{g}_{\mathbf{d}} | \cdot\right)=MVN(\boldsymbol{\mu}_{\mathbf{d}}\mathbf{,}\boldsymbol{\Sigma}_{\mathbf{gd}})$

$$\boldsymbol{\Sigma}_{\mathbf{gd}}={(\frac{1}{4}{\mathbf{D}^{\boldsymbol{*}}}^{\mathbf{T}}\left( \boldsymbol{\Sigma} \right)^{\mathbf{-1}}\mathbf{D}^{\boldsymbol{*}}\mathbf{+}{\mathbf{C}_{\mathbf{d}}}^{\mathbf{-1}})}^{-1}$$

$$\boldsymbol{\mu}_{\mathbf{d}}=\frac{1}{2}\boldsymbol{\Sigma}_{\mathbf{gd}}{\mathbf{D}^{\boldsymbol{*}}}^{\mathbf{T}}\left( \boldsymbol{\Sigma} \right)^{\mathbf{-1}}\mathbf{(y-}\mathbf{X}^{\boldsymbol{*}}\mathbf{b-}\frac{1}{2}\mathbf{S}^{\boldsymbol{*}}\mathbf{g}_{\mathbf{s}}\mathbf{)}$$

$$P\left( \mathbf{b} | \boldsymbol{\cdot} \right)\sim MVN(\boldsymbol{\mu}_{\mathbf{b}}\mathbf{,}\boldsymbol{\Sigma}_{\mathbf{b}}\boldsymbol{)}$$

$$\boldsymbol{\Sigma}_{\mathbf{b}}\boldsymbol{=}{{\mathbf{(}\mathbf{X}^{\boldsymbol{*}}}^{\mathbf{T}}\left( \boldsymbol{\Sigma} \right)^{\mathbf{-1}}\mathbf{X}^{\boldsymbol{*}}\mathbf{+}{{(V}_{b}\mathbf{I}_{\mathbf{n}}\boldsymbol{)}}^{-1})}^{\boldsymbol{-1}}$$

$$\boldsymbol{\mu}_{\mathbf{b}}\boldsymbol{=}\boldsymbol{\Sigma}_{\mathbf{b}}\left( \boldsymbol{\Sigma} \right)^{\mathbf{-1}}\mathbf{(y-}\frac{1}{2}\mathbf{S}^{\boldsymbol{*}}\mathbf{g}_{\mathbf{s}}\mathbf{-}\frac{1}{2}\mathbf{D}^{\boldsymbol{*}}\mathbf{g}_{\mathbf{d}}\mathbf{)}$$

In order to choose the most appropriate number of basis functions, we ran the full analysis using 2, 4, 6, 7 and 8 basis functions. The estimated genetic variance-covariance matrices are shown in Fig. S1. We also calculated the Akaike Information Criteria (AIC) under each model and summarize the results in Fig. S1.

*Importance resampler*

Once the posterior distribution of $\boldsymbol{\Sigma}$, $\mathbf{C}_{\mathbf{s}}$ and $\mathbf{C}_{\mathbf{d}}$ are obtained via Gibbs sampling (described above), importance weights were calculated for each Gibbs sample to account for family size differences across time. Specifically,

$w_{f}=\prod_{t} w_{f,t}=\prod_{t} \frac{L\left( \mathbf{y} | \mathbf{g}_{\mathbf{s}}\mathbf{,}\mathbf{g}_{\mathbf{d}},\mathbf{ℇ},N_{c} \right)}{L\left( \mathbf{y} | \mathbf{g}_{\mathbf{s}}\mathbf{,}\mathbf{g}_{\mathbf{d}},\mathbf{ℇ},N \right)}$

where ${N_{c}}_{t(i,j)}=\left\{ \begin{matrix} \frac{1}{N_{t(i,j)}} where i=j \\ \frac{1}{N_{t(max)}} where i\neq j \end{matrix} \right.$ .

Then for each Gibbs iteration, $w_{iter}=\prod_{f} w_{f}$. The log of the weight was used for numerical stability. All of the weights $w_{iter}$ were then normalized to the largest $w_{iter}$.

Not all of the families survived the entire experiment period. In order to make full use of all the data collected, the missing values are drawn in each Gibbs step conditional on the present draw of parameters. Specifically, $\boldsymbol{y=[}\boldsymbol{y}_{\boldsymbol{1}} \boldsymbol{y}_{\boldsymbol{2}}\boldsymbol{]}$ for a particular family and $\boldsymbol{y}_{\boldsymbol{2}}$ is the unobserved data. Let the mean for the entire vector be $\boldsymbol{\mu}_{\boldsymbol{y}}=[\boldsymbol{\mu}_{\boldsymbol{1}}\boldsymbol{\mu}_{\boldsymbol{2}}]$ and the variance-covariance matrix $\boldsymbol{\Sigma}_{\boldsymbol{y}}=\left[ \begin{matrix} \boldsymbol{\Sigma}_{\boldsymbol{11}} & \boldsymbol{\Sigma}_{\boldsymbol{12}} \\ \boldsymbol{\Sigma}_{\boldsymbol{21}} & \boldsymbol{\Sigma}_{\boldsymbol{22}} \end{matrix} \right]$, then the conditional distribution for $\boldsymbol{y}_{\boldsymbol{2}}$ is given by multivariate normal distribution with $\boldsymbol{\mu}_{\boldsymbol{2|1}}\boldsymbol{=}\boldsymbol{\mu}_{\boldsymbol{1}}\boldsymbol{+}\boldsymbol{\Sigma}_{\boldsymbol{12}}{\boldsymbol{\Sigma}_{\boldsymbol{11}}}^{\boldsymbol{-1}}\boldsymbol{(}\boldsymbol{y}_{\boldsymbol{1}}\boldsymbol{-}\boldsymbol{\mu}_{\boldsymbol{1}}\boldsymbol{)}$ and $\boldsymbol{\Sigma}_{\boldsymbol{2|1}}\boldsymbol{=}\boldsymbol{\Sigma}_{\boldsymbol{22}}\boldsymbol{-}\boldsymbol{\Sigma}_{\boldsymbol{12}}{\boldsymbol{\Sigma}_{\boldsymbol{11}}}^{\boldsymbol{-1}}{\boldsymbol{\Sigma}_{\boldsymbol{12}}}^{\boldsymbol{T}}$**.**

*S2. Convergence assessment*

The effective sample size is defined as the $\frac{sample size}{(1+var (weight))}$ (Liu 2001) and can be approximated as $\frac{{(\sum weight)}^{2}}{\sum{weight}^{2}}$, which is 94% of the total Gibbs samples. Different starting values were used to assess the convergence. Specifically, 5 independent chains were run for 10000 iterations and reached the same final posterior distribution.

Table S1 Model comparison of models where the number of basis ranged between 2 to 8 using Akaike information criterion (AIC). The model with the lowest AIC is marked with *.

| Basis number | mean(lnL) | AIC |
| --- | --- | --- |
| 2 | -130540 | 262989 |
| 4 | 104650 | -206567 |
| 5 | 220600 | -438055 |
| 6 | 221110 | -438663 |
| 7* | 223570 | -443171 |
| 8 | 212430 | -420479 |

Fig. S1


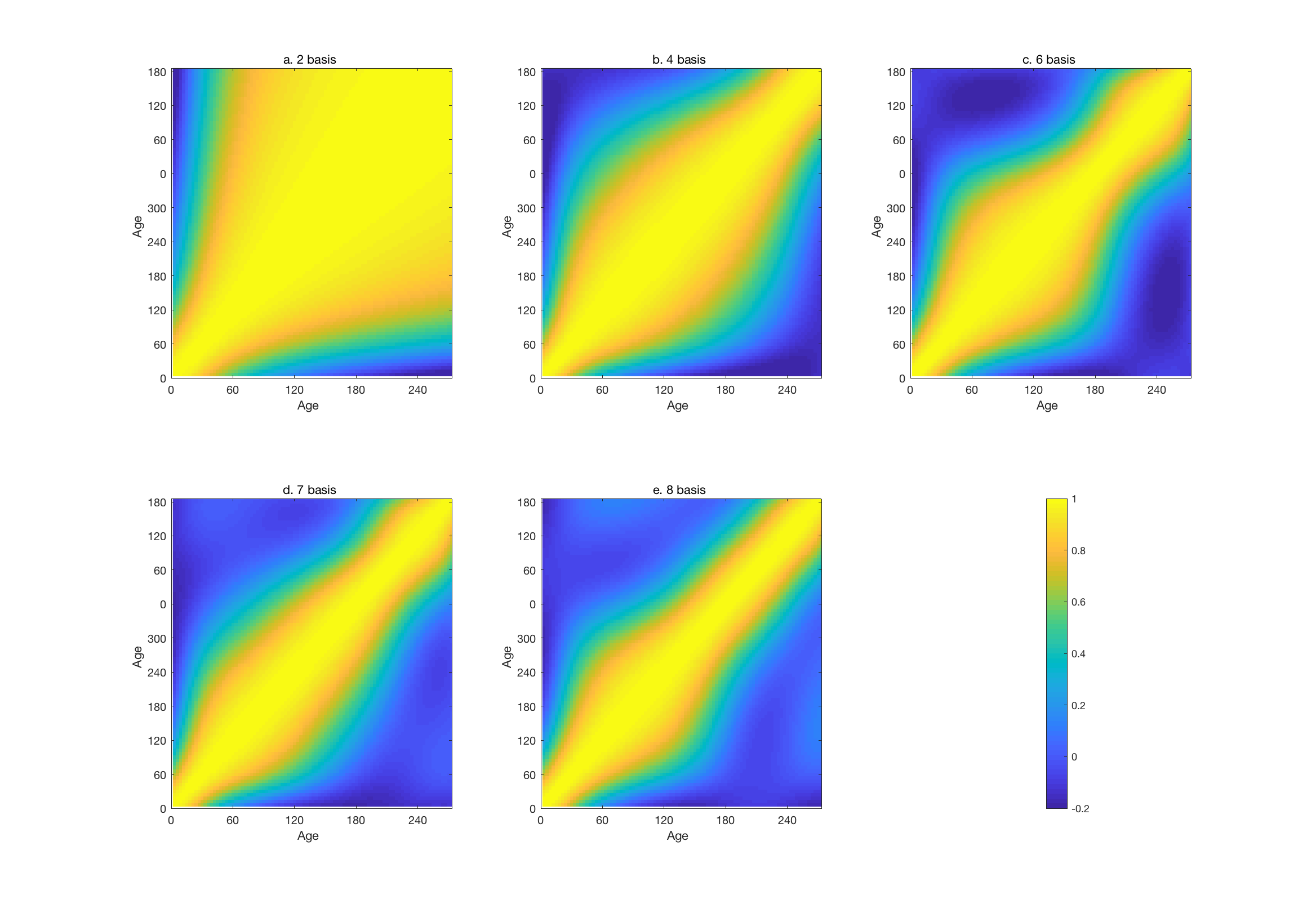


Fig. S2


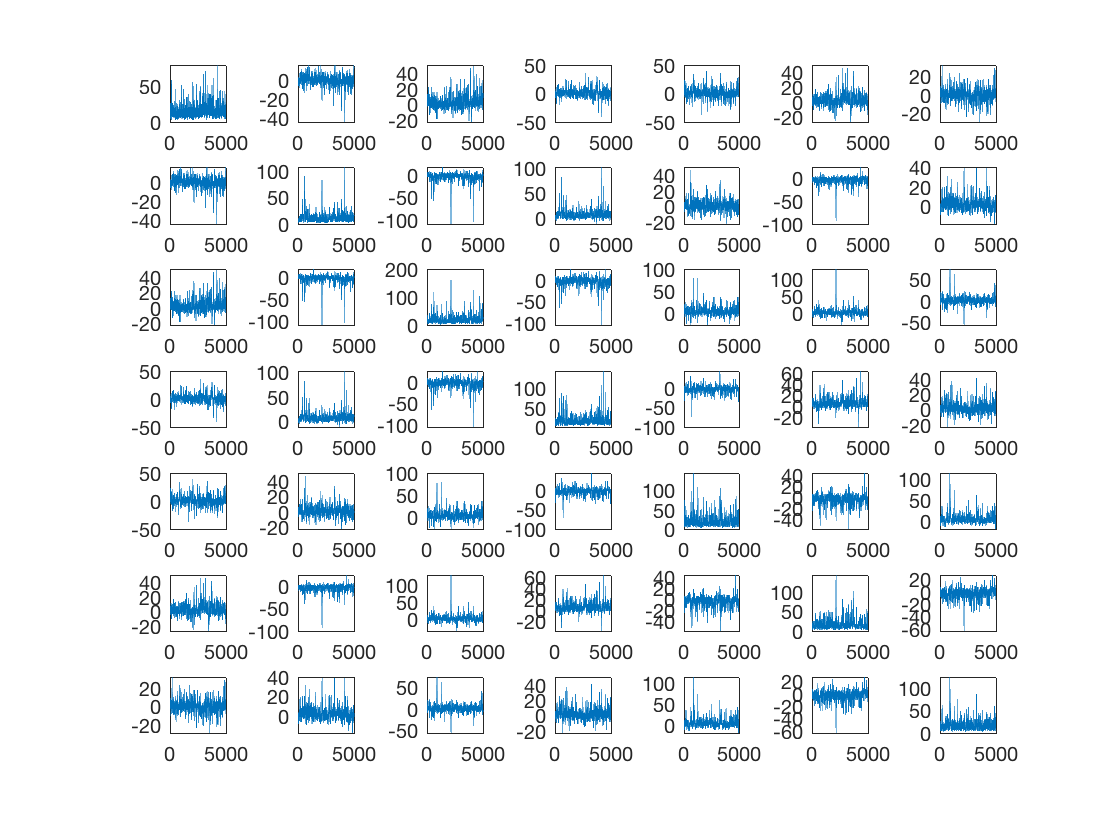

Supplement: Supplementary file 1 [file EVA-12-964-s001.docx]
